# Differences in surface chemistry of iron oxide nanoparticles result in different routes of internalization

**DOI:** 10.3762/bjnano.12.22

**Published:** 2021-03-23

**Authors:** Barbora Svitkova, Vlasta Zavisova, Veronika Nemethova, Martina Koneracka, Miroslava Kretova, Filip Razga, Monika Ursinyova, Alena Gabelova

**Affiliations:** 1 Cancer Research Institute, Biomedical Research Center of the Slovak Academy of Sciences, Dubravska cesta 9, 845 05 Bratislava, Slovakia; 2 Institute of Experimental Physics, Slovak Academy of Sciences, Watsonova 47, 040 01 Kosice, Slovakia; 3 Faculty of Medicine, Comenius University, Spitalska 24, 813 72 Bratislava, Slovakia; 4 Selecta Biotech SE, Istrijska 20, 841 07 Bratislava, Slovakia; 5 Slovak Medical University, Limbova 12, 833 03 Bratislava, Slovakia

**Keywords:** bovine serum albumin, cellular uptake, magnetic iron oxide nanoparticles, polyethylene glycol, surface coating

## Abstract

The efficient entry of nanotechnology-based pharmaceuticals into target cells is highly desired to reach high therapeutic efficiency while minimizing the side effects. Despite intensive research, the impact of the surface coating on the mechanism of nanoparticle uptake is not sufficiently understood yet. Herein, we present a mechanistic study of cellular internalization pathways of two magnetic iron oxide nanoparticles (MNPs) differing in surface chemistry into A549 cells. The MNP uptake was investigated in the presence of different inhibitors of endocytosis and monitored by spectroscopic and imaging techniques. The results revealed that the route of MNP entry into cells strongly depends on the surface chemistry of the MNPs. While serum bovine albumin-coated MNPs entered the cells via clathrin-mediated endocytosis (CME), caveolin-mediated endocytosis (CavME) or lipid rafts were preferentially involved in the internalization of polyethylene glycol-coated MNPs. Our data indicate that surface engineering can contribute to an enhanced delivery efficiency of nanoparticles.

## Introduction

Magnetic iron oxide nanoparticles (MNPs) as chemically inert material have been increasingly employed as contrast agents in magnetic resonance imaging (MRI), positron emission tomography (PET), and near-infrared fluorescence (NIRF) imaging [1]. The superparamagnetic properties of MNPs make them eligible for the targeted delivery of the drug-loaded particles to the tumor mass via an external magnetic field [2]. Furthermore, MNPs are promising biosensors [3] and antimicrobial tools [4], and they play an important role in the development of multifunctional theranostics to combat cancer [5]. MNPs are easily manufactured and biocompatible. Also, there are physiologically well tolerated as iron is an essential nutrient for almost all life forms [6]. Iron oxide nanoparticles are the only one FDA-approved magnetic nanoparticles for biomedical application (Resovist).

Efficient cellular internalization of nanoparticles is one of the critical steps during the development of new nanotechnology-based pharmaceuticals. Nanoparticles can enter the cell through various specific and non-specific pathways of endocytosis, divided into two broad categories, that is, phagocytosis and pinocytosis [7]. While phagocytosis occurs primarily in specialized cells, such as macrophages or monocytes, other endocytic pathways occur in virtually all cells [8]. Clathrin-mediated endocytosis (CME) is the predominant endocytosis pathway and is involved mainly in nutrient intake and intracellular communication [9–10]. CME is initiated by the polymerization of clathrin units resulting in the assembly of a basket-like structure (clathrin-coated pit) with a size of 120–150 nm at the inner layer of the plasma membrane [11]. An alternative endocytosis pathway is caveolin-mediated endocytosis (CavME), the second most important route of cellular entry. Caveolae are characteristic flask-shaped membrane invaginations with an average size of 50–100 nm [12], lined by caveolin and enriched with cholesterol and sphingolipids. The deeply invaginated clathrin or caveolin pits are then fissured from the membrane by GTPase dynamin. Macropinocytosis, a clathrin- and caveolin-independent endocytosis pathway, occurs via actin-driven membrane protrusions. The large endocytic vesicle has a size bigger than 1 µm [13]. Alternative pathways of endocytosis involve other types of cholesterol-rich microdomains called “lipid rafts”, small structures of 40–50 nm in diameter [14], or glycosylphosphatidylinositol (GPI)-anchored proteins [15].

Inorganic nanoparticles are frequently engineered with an organic surface coating to improve their biocompatibility, colloidal stability, and bioavailability. Moreover, the coating facilitates their further functionalization to increase their accumulation in the tumor mass [3]. Numerous coating moieties have been employed to modify the surface properties of MNPs [16]. Among them, polyethylene glycol (PEG), a non-degradable polyether of the monomer ethylene glycol, and bovine serum albumin (BSA), a versatile protein carrier, are the most frequently used materials for biomedical applications [17–20]. The impact of the surface chemistry on the mechanism of nanoparticle uptake has not been sufficiently clarified yet.

MNPs with comparable basic physicochemical characteristics (e.g., particle size, surface charge, and magnetism) differing only in surface modification, BSA-SO-MNPs and PEG-SO-MNPs, have been synthesized to study the effect of surface modification on cellular internalization. Human lung A549 cells were selected as a model system to investigate the uptake of surface-modified MNPs. These cells are a valuable in vitro model of human alveolar epithelial type-2 cells [21], which are considered as drivers of lung fibrosis [22] and lung tumor development [23]. Inhalation therapy represents a prospective non-invasive curative modality for lung cancer and a therapy for other lung illnesses [24]. Drug delivery through the inhalation of nanoparticles is a promising treatment modality against lung cancers conferring high pulmonary drug concentrations while minimizing the side effects [25].

The internalized amount of the tested MNPs in A549 cells in the presence of compounds that inhibit either endocytosis or cytoskeleton dynamics was quantified by atomic absorption spectroscopy (AAS) and the uptake was verified by fluorescent microscopy. The uptake route of the tested MNPs differed depending on the surface coating. While BSA-SO-MNPs were internalized via CME, PEG-SO-MNPs were preferentially taken up through CavME or lipid rafts. Co-localization studies confirmed the entrapment of fluorescently labeled RITC-BSA-SO-MNPs in clathrin-coated vesicles in A549 cells stably expressing green fluorescent protein (GFP)-clathrin. The results indicate that tuning of the MNP surface chemistry can potentially provide delivery strategies featuring enhanced targeting.

## Results

### Expression of the key proteins involved in the endocytosis in A549 cells

The determination of the proficiency/deficiency of cells regarding key proteins involved in specific endocytic pathways is essential before defining the route of cellular uptake of nanoparticles. Therefore, initial experiments were focused on the expression of clathrin heavy chain (CLHC), dynamin (Dyn), caveolin-1 (Cav1), and its phosphorylated form (pCav1) in A549 cells. The expression of CLHC and Cav1 was determined at the protein (Supporting Information File 1, Figure S1A) and the mRNA level (Supporting Information File 1, Figure S1B). The expression of Dyn was analyzed only at the protein level. Our results demonstrated that A549 cells are proficient in both CME and CavME pathways.

### The effect of endocytic inhibitors on cell proliferation and morphology

The experiments with the positive controls confirmed the ability of endocytic inhibitors to block the specific route of endocytosis. None of the endocytic inhibitors affected cell viability and proliferation activity with the exception of nocodazole (Noc) (Supporting Information File 1, Figure S2). Short-term exposure of cells to surface-modified MNPs and Noc affect substantially the cell proliferation and morphology. Noc affects microtubule formation, thus interfering with cytoskeleton structure and mitosis, leading to cell cycle arrest in G2/M [26]. As MNPs interfere with tubulin polymerization as well [27], the combined treatment could multiply the microtubule-disrupting effect of Noc.

### The effect of endocytic inhibitors on the internalization of BSA-SO-MNPs and PEG-SO-MNPs

**Figure 1:**
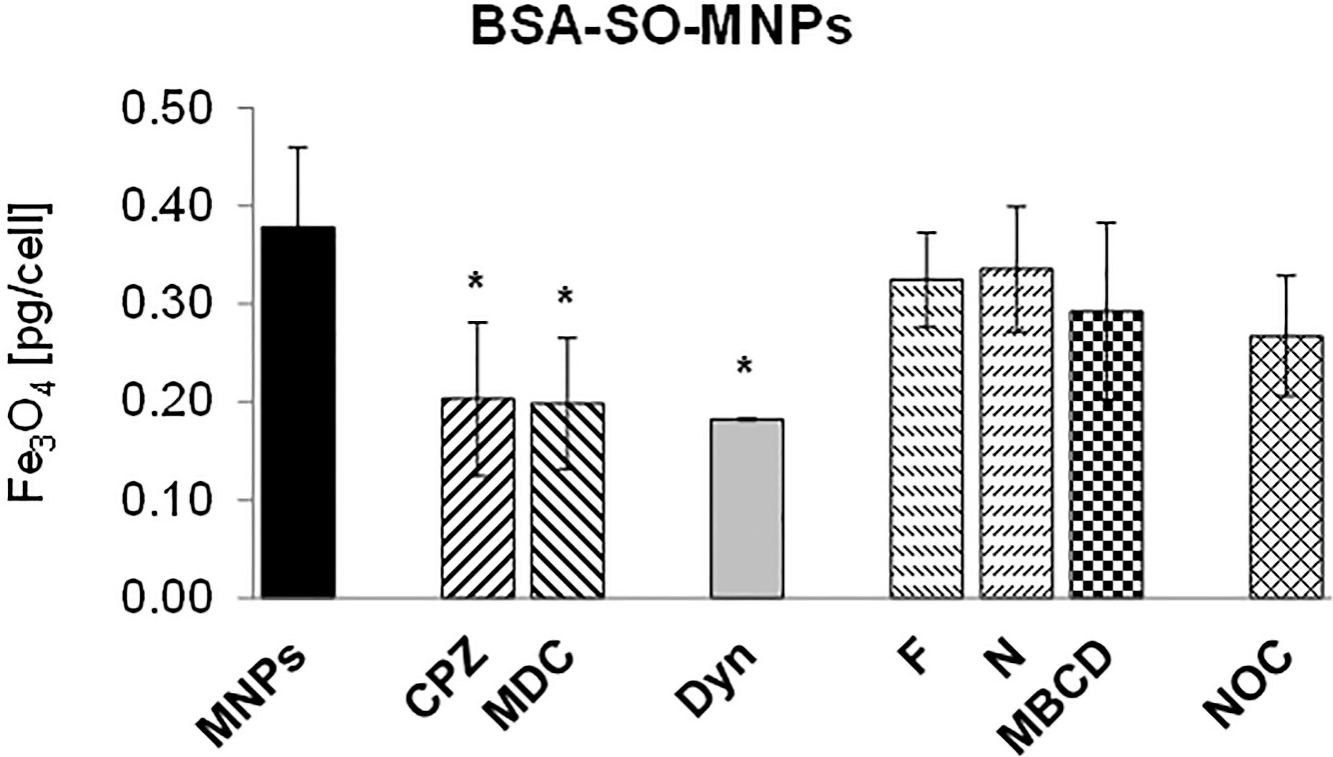
The effect of endocytic inhibitors on the internalization of BSA-SO-MNPs. MNPs – BSA-SO-MNPs (2 mM), CPZ – chlorpromazine (25 µM), MDC – monodansylcadaverine (150 µM), Dyn – dynasore (80 µM), F – filipin (5 µM), N – nystatin (20 µM), MBCD – methyl-β-cyclodextrin (100 uM), NOC – nocodazole (25 µM). Data are given as mean values ± SD from at least three independent experiments carried out twice.

In the absence of inhibitors, differences in the internalized amount of surface-modified MNPs in A549 cells were found. PEG-SO-MNPs were taken up by A549 cells more efficiently than BSA-SO-MNPs (8.3 pg/cell vs 0.39 pg/cell, respectively). The impact of inhibitors on the internalization of BSA-SO-MNPs is shown in Figure 1. A significant decrease in the internalized amount of BSA-SO-MNPs was quantified in cells treated with CME inhibitors, that is, chlorpromazine (CPZ), monodansylcadaverine (MDC), and dynasore (Dyn). Only negligible changes in BSA-SO-MNP uptake were found in cells treated with CavME inhibitors, that is filipin (F), nystatin (N), and methyl-β-cyclodextrin (MBCD). A slight reduction in the internalized amount of BSA-SO-MNPs was also detected in cells treated with Noc but the decrease did not reach significance.

**Figure 2:**
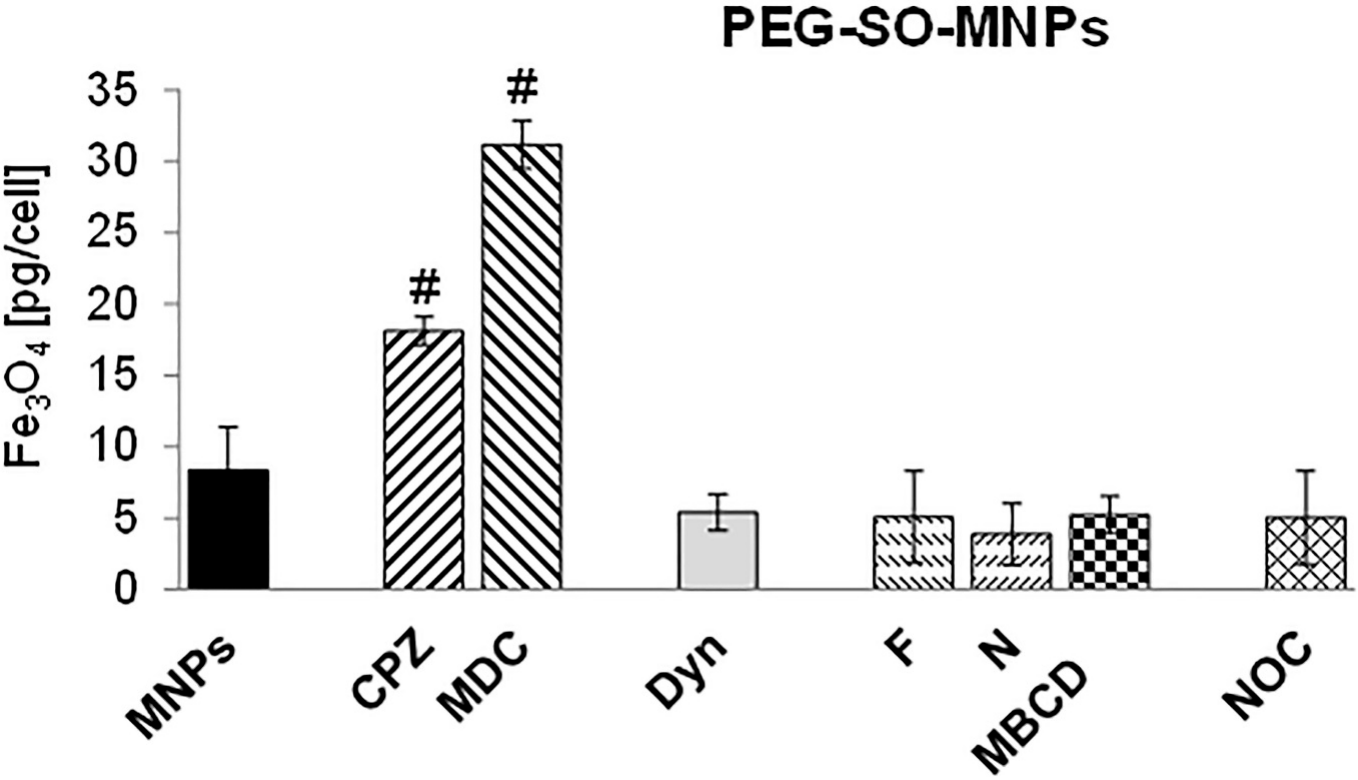
The effect of endocytic inhibitors on the internalization of PEG-SO-MNPs. MNPs – PEG-SO-MNPs (2 mM), CPZ – chlorpromazine (25 µM), MDC – monodansylcadaverine (150 µM), Dyn – dynasore (80 µM), F – filipin (5 µM), N – nystatin (20 µM), MBCD – methyl-β-cyclodextrin (100 uM), NOC – nocodazole (25 µM). Data are given as mean values ± SD from at least two independent experiments carried out twice.

In contrast, a substantial reduction in the internalized amount of PEG-SO-MNPs was detected in the presence of CavME and lipid-raft inhibitors (Figure 2). These results indicated that PEG-SO-MNPs entered into A549 cells via CavME or another CME-independent route of endocytosis. Surprisingly, the inhibition of CME by CPZ and MDC resulted in a significantly increased uptake of PEG-SO-MNPs compared to control cells.

### Positive controls

A fluorescently labeled Alexa Fluor 594–transferrin conjugate (Tr), which enters the cell via CME [28] and a cholera toxin B subunit–fluorescein isothiocyanate (FITC) conjugate (ChT), the internalization of which is mediated via CavME [29], were used as positive controls to test the ability of inhibitors to affect particular endocytic pathways (Supporting Information File 1, Figure S3). Image analysis revealed a blocking of Tr internalization after cell exposure to CPZ and MDC, inhibitors of CME, although the effect of MDC was less pronounced. A substantial reduction of ChT internalization was found after cell exposure to F and N, inhibitors of CavME; the effect of F was higher than that of N. Interestingly, MBCD could neither inhibit the cellular internalization of Tr nor that of ChT. Application of Dyn resulted in the accumulation of Tr as well as that of ChT in larger spots, probably in the clathrin-coated pits/caveolae at the inner side of the membrane that might not be pinched off. No distinct changes in Tr or ChT uptake were observed in cells exposed to Noc.

### Co-localization study

**Figure 3:**
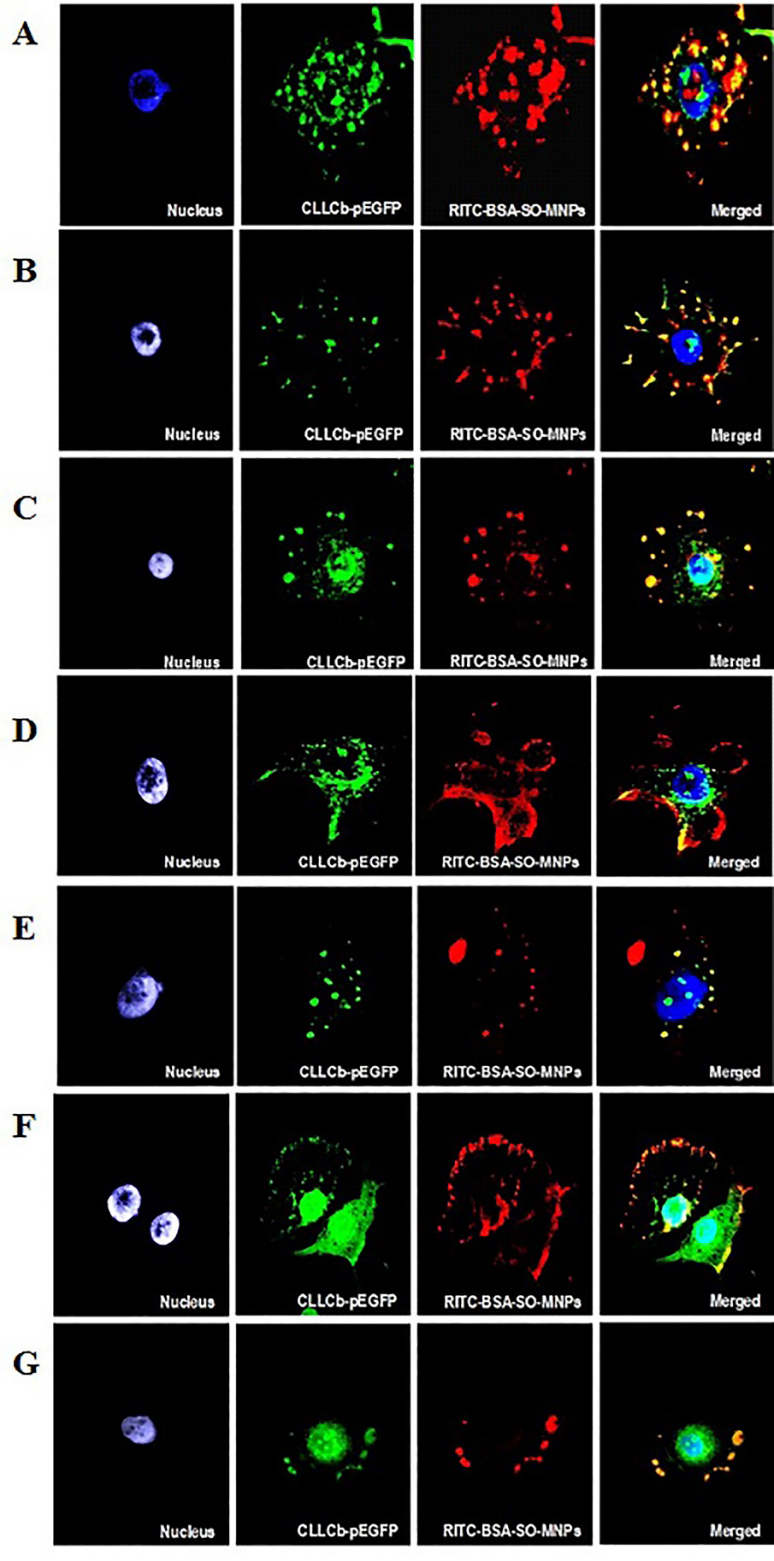
The effect of endocytic inhibitors or cytoskeleton dynamics inhibitors on RITC-BSA-SO-MNPs internalization. A – control, B – chlorpromazine, C – monodansylcadaverine, D – dynasore, E – methyl-β-cyclodextrin, F – filipin, G – nystatin. Blue – nucleus (DAPI), green – clathrin, red – RITC-BSA-SO-MNPs, merged; magnification 630×.

To avoid misinterpretation of results obtained from AAS because of the relatively low uptake of BSA-SO-MNPs into A549 cells (0.39 pg/cell) fluorescently labeled rhodamine B isothiocyanate (RITC)-BSA-SO-MNPs were synthesized. Furthermore, to verify the assumed route of entry and cellular localization of BSA-SO-MNPs, genetically engineered A549 cells stably expressing FITC–clathrin were prepared (Supporting Information File 1, Figure S4). In the absence of endocytic inhibitors, the red signal of RITC-BSA-SO-MNPs co-localized with the green signal of FITC–clathrin resulted in a yellow signal. The images from fluorescent microscopy indicated the uptake of RITC-BSA-SO-MNPs via CME. In the presence of CPZ and MDC, which are CME inhibitors, the yellow signal (co-localization) or the red signal (corresponding to free RITC-BSA-SO-MNPs) were mainly localized at the cell membrane while clathrin (green signal) was detected in the cytoplasm (Figure 3B and Figure 3C). These results confirmed the dominant role of CME in the internalization of BSA-coated MNPs. In the presence of Dyn, the signal of RITC-BSA-SO-MNPs and FITC–clathrin was co-localized on the cell membrane (yellow signal), indicating the accumulation of a fraction of nanoparticles in the clathrin-coated pits (Figure 3D). The pattern of the co-localization signal of RITC-BSA-SO-MNPs and FITC–clathrin implied that the lipid-raft inhibitor might also affect the CME uptake (Figure 3E). In contrast, the application of CavME inhibitors did not substantially affect the internalization of RITC-BSA-SO-MNPs into A549 cells (Figure 3F and Figure 3G). No reduction in the co-localization pattern of RITC-BSA-SO-MNPs and FITC–clathrin was measured in A549 cells exposed to CavME inhibitors (i.e., F and N).

## Discussion

The internalization of nanoparticles is a dynamic energy-dependent and highly regulated process, affected by physicochemical characteristics of nanoparticles (e.g., shape, size, surface chemistry, and surface charge), cell membrane properties (fluidity, type of receptors, and receptor density), and cell type [30–32]. For biomedical applications, the optimal size of nanocarriers is in the range of 95–200 nm because of the higher accumulation rate in tumors [33–34]. Spherical nanoparticles (NPs) in the range of 100–200 nm have been shown to extravagate through vascular fenestrations of tumors (the EPR effect) and escape filtration by liver and spleen [35]. Nanoparticles smaller than 10 nm can be easily cleared by physiological systems (filtration through the kidney), while particles larger than 200 nm may be cleared by phagocytic cells in the reticulo-endothelial system (RES). Many studies reported that therapeutic nanoparticles in the size range of 20–200 nm showed a higher accumulation rate in tumors because they cannot be recognized by the RES nor filtrated by the kidney [33,36]. In case of a pulmonary disease, where the airway–mucus barrier is difficult to penetrate, nanoparticles in the size range of 200 nm are more effective in mucus penetration [20,37]. The effect of surface chemistry on the mechanism of NPs uptake is, however, not sufficiently understood yet. Understanding the distinct pathway(s) underlying cellular uptake and the parameters influencing this process is of great importance for the design of new tailored nanovectors for different cells/tissues in biomedical applications.

It is well documented that the physicochemical parameters of nanoparticles substantially affect the uptake. Once NPs enter biological fluids (blood or culture medium with serum), proteins immediately adsorb onto the surface of the NPs, forming a layer called protein corona (PC). The PC changes the surface composition and structure of NPs, directly influences the cell–NP interactions, determines the toxicity of NPs, and affects geometry and size of NPs, which play a crucial role in cellular uptake [38–39]. Despite nearly identical magnetite core size, hydrodynamic size, and zeta potential of the MNPs in the stock solution, the hydrodynamic size of PEG-SO-MNPs in culture medium was almost three times that of BSA-SO-MNPs (281 nm vs 98 nm, respectively) due to absorption of serum proteins on the particle surface [40]. Although PEGylation decreases the protein absorption on the particle surface, it does not completely prevent it [41]. BSA is a component of serum proteins and commonly involved in PC formation. Hence, coating of MNPs with BSA can be considered as a PC per se. As a dysopsonin protein, albumin promotes a prolonged blood circulation time through blocking the recognition by macrophages [42]. A comprehensive characterization of nanoparticles in biological fluids is, therefore, essential for the interpretation of their biological effects, including cellular uptake [43]. Nevertheless, PEG-SO-MNPs were more efficiently internalized into A549 cells than BSA-SO-MNPs. Interestingly, the magnetic nanospheres (PEG-SO-MNPs coated with polylactic-*co*-glycolic acid, PLGA) were taken up by A549 cells to the same extent as BSA-SO-MNPs, even though their hydrodynamic particle size was more than five times larger [44]. A less efficient uptake of BSA-coated MNPs compared to PEG-coated MNPs has been observed also in primary murine podocytes [45]. Superparamagnetic iron oxide nanoparticles covered with BSA were shown to possess a lower affinity to the cellular membrane [42]. This lower level of “stickiness” to the cellular membrane could explain the low uptake into A549 cells compared to PEG-SO-MNPs. The surface chemistry of nanoparticles was shown to largely determine the composition of the protein corona in terms of the amount and specificity of proteins adsorbed from the serum, overall affecting the final size of the nanoparticles in the biological fluid [46].

Because the cellular entry mechanism of identical nanoparticles can differ between cancer cells and non-malignant cells [47], only one cell line, human lung A549 cells, proficient in clathrin and caveolin, were employed. Kuhn et al. [48] confirmed the proficiency of A549 cells regarding these key proteins involved in specific endocytic pathways and demonstrated their presence at the cell membrane and in the cytoplasm.

To probe the mechanism involved in NP uptake, various inhibitors of endocytosis with a different mechanism of action are frequently used [49]. CPZ causes a loss of clathrin from the cell surface by inhibiting the function of adaptor protein 2 (AP2), one of the key adaptor proteins in clathrin-mediated endocytosis (CME). MDC, a competitive inhibitor, blocks the enzyme transglutaminase 2, which is necessary for receptor crosslinking in the region of clathrin-coated pits [48]. Consistent with our results, Rothen-Rutishauser et al. [50] and Francia et al. reported that CPZ was more efficient in blocking transferrin (Tr) internalization than MDC in A549 cells. Caveolae and lipid raft internalizations are known to be inhibited by N, F, and MBCD through depletion of cholesterol from the cell membrane [51]. While F and N were described to be very specific inhibitors of caveolin-mediated endocytosis (CavME) without affecting CME and micropinocytosis [50], MBCD is not so specific. It can affect components of the transport machinery involved in multiple endocytic pathways including CME, depending on the concentration used [51]. F binds cholesterol within membranes while N perturbs cholesterol levels by inhibition of de novo synthesis [52]. While both F and N blocked the internalization of cholera toxin (ChT) into A549 cells, no effect of MBCD on ChT uptake was observed. Similar results were obtained in J774A.1 macrophages [48]. In contrast, Rothen-Rutishauser et al. [50] did not find any inhibition of ChT uptake after exposure of A549 cells to N or F and only a partial impairment of Tr uptake in the presence of MBCD. Dyn, a cell-permeable small molecule that inhibits GTPase activity of dynamin, acts fast, that is, within seconds. Dynamin is essential for clathrin-coated vesicle formation as well as for ligand uptake through caveolae [53].

A substantial lower amount of BSA-SO-MNPs was internalized in A549 cells in the presence of CME inhibitors and Dyn compared to the control. However, no differences between CPZ and MDC in their capacity to inhibit the internalization of BSA-SO-MNPs were observed. In contrast, none of the CavME inhibitors reduced significantly the entry of BSA-SO-MNPs into A549 cells. CME as the predominant entry pathway of BSA-SO-MNPs into A549 cells was confirmed quantitatively (by AAS) as well as qualitatively (by image analysis), two standard methods commonly utilized to monitor the uptake of nanomaterials [54]. In agreement with our results, Yumoto et al. [55] also determined the predominant uptake of fluorescently labeled albumin via CME in A549 cells. The authors supposed that macropinocytosis but not CavME may also be involved in albumin internalization. Similarly, the internalization of BSA-SO-MNPs via CME was also observed in murine primary podocytes and mesangial cells (unpublished results). In endothelial cells, albumin has been shown to bind to albondin, a 60 kDa glycoprotein (gp60) receptor localized in caveolae. The interaction between gp60 and caveolin-1, which is upregulated in many cancer types [56] aids the vesicle formation, facilitating the accumulation of albumin in the tumor mass [57]. This phenomenon forms the basis of Abraxane, a 130 nm paclitaxel-bound albumin nanodrug [58]. There is, however, no evidence that albondin is expressed on tumor cells; in contrast, albumin-binding proteins are present on various human tumor cell lines derived from solid tumors [59].

In contrast to BSA-SO-MNPs, CavME was the predominant mechanism involved in the uptake of PEG-SO-MNPs. The internalized quantity of PEG-SO-MNPs was substantially lower compared with control cells when F, N, MBCD, or Dyn were added to the culture medium. Similar to our results, Brandenberger et al. [60] also observed a reduced internalization of PEG-coated gold nanoparticles after exposure to the endocytic inhibitor MBCD.

As the hydrodynamic size in the culture medium was increased (for BSA-SO-MNPs from 70 to 98 nm and for PEG-SO-MNPs from 76 to 281 nm) this phenomenon might contribute to different cell uptake pathways as well. Inhibiting CME resulted in the preferential uptake of smaller NPs (smaller than 200 nm) into cells via CME. Larger NPs (larger than 200 nm) were internalized by CavME [29], just like in our study. In line with our results, Langston Suen and Chau [61] reported the uptake of folate-decorated NPs of 50 nm in size by CME into retinal pigment epithelium cells, while 250 nm particles were dominated by CavME.

Surprisingly, the addition of CME inhibitors to the culture medium increased the uptake of PEG-SO-MNPs. CME is the major endocytic pathway in mammalian cells; about 95% of all molecules internalized into cells are taken up via clathrin-coated pits [62]. It is reasonable to suppose that inhibition of this pathway by CME inhibitors, on one hand, and saturation of clathrin-independent endocytic pathways by exposure to PEG-SO-MNPs, on the other hand, could compromise cellular homeostasis and result in cellular stress. A close relation between endocytosis and cellular stress has been highlighted in several publications [63]. We can hypothesize that under such conditions either different endocytic pathways, for example, macropinocytosis as suggested by Rothen-Rutishauser et al. [50] or non-selective alternative endocytic structures, discussed by Boucrot et al. [64] or Vega et al. [65], could be upregulated to provide compensatory endocytic pathways. Unfortunately, the regulation of CME under different physiological conditions is poorly understood and other studies are required to bridge this gap in our knowledge.

## Conclusion

MNPs coated with bovine serum albumin (BSA-SO-MNPs) and polyethylene glycol (PEG-SO-MNPs), were found to enter the cells by different routes of endocytosis. BSA-SO-MNPs were internalized via CME while PEG-SO-MNPs were taken up via CavME or lipid rafts. These findings confirm the major role of nanoparticle coatings on cellular entry mechanisms. Our data suggest that the effects of endocytic inhibitors on the internalization pathways are rather complex. MNPs may be internalized by several endocytic pathways simultaneously, although with varying efficiency, and inhibition of one endocytic pathway can subsequently stimulate other routes of their internalization. Understanding the mechanisms of cellular uptake is of particular importance for the design of new nanotechnology-based pharmaceuticals and their targeting to specific intracellular locations.

## Experimental

### Magnetic iron oxide nanoparticles (MNPs)

Synthesis, coating, and physicochemical characteristics of the spherical magnetic iron oxide nanoparticles with magnetite (Fe_3_O_4_) core and different hydrophilic shells have been published before [37]. Two types of MNPs were used in this study: BSA-SO-MNPs (weight ratio of BSA/magnetite = 2) and PEG-SO-MNPs (weight ratio of PEG/magnetite = 0.25). Additionally, RITC-BSA-SO-MNPs (RITC/BSA, ratio 4:1) were used in some experiments. The basic characteristics of these nanoparticles in the solvent and culture medium are shown in Table 1.

**Table 1:** Basic physicochemical properties of surface-modified magnetite nanoparticles.

Quantity	BSA-SO-MNPs	PEG-SO-MNPs

magnetite inner core diameter [nm]	10.09 ± 0.11	9.92 ± 0.16
particle size (*D*_H_) diameter in H_2_O [nm]	70.0 ± 3.49	76.0 ± 2.52
*I*_s_ at 295 K [Am^2^kg^−1^]	2.07 ± 0.01	2.17 ± 0.01
*M*_sat_ [mT]	2.74 ± 0.01	2.87 ± 0.04
zeta potential (ζ) [mV]	−48 ± 0.3	−44 ± 0.5
surface modification, *M*_w_ [g/mol]:		
C_18_H_33_NaO_2_	304.44	304.44
bovine serum albumin (BSA)	66,000	—
polyethylene glycol (PEG)	—	1,000
ratio (BSA, PEG)/Fe_3_O_4_	2	0.25
PDI	0.16 ± 0.01	0.12 ± 0.01
particle size distribution and mean diameter in culture medium [nm]	98.0 ± 8.0 unimodal	281 ± 4.0 unimodal
PDI	0.14 ± 0.01	0.17 ± 0.01
zeta potential (ζ) in culture medium [mV]	−16.3 ± 0.9	−14.1 ± 0.9

### Dynamic light scattering (DLS)

Particle size distribution and zeta potential of the surface-modified MNPs in stock solution and culture medium were determined by DLS using a Zetasizer Nano-ZS (Malvern Instruments, UK) equipped with a 4 mW helium/neon laser (λ = 633 nm) and a thermoelectric temperature controller at 37 °C. The characteristics of nanoparticles and culture medium have been published before [66–67].

### Cell line

A549 (ATCC^®^ CCL-185™) cells were obtained from LambdaLife (Bratislava, Slovakia). The cells were maintained in Dulbecco’s Modified Eagle’s Medium (DMEM) supplemented with 10% fetal bovine serum (FBS) and antibiotics (penicillin, 100 U/mL; streptomycin and kanamycin, 100 µg/mL). Cells seeded at a density of 2 × 10^3^ cm^−2^ to 1 × 10^4^ cm^−2^, were cultured in a humidified atmosphere of 5% CO_2_ at 37 °C. In all experiments, cells were exposed to MNPs in a medium supplemented with 2% FBS. A549 cells up to 20 generations (passaged two times per week) were used in the experiments.

### Treatment of cells

**Figure 4:**
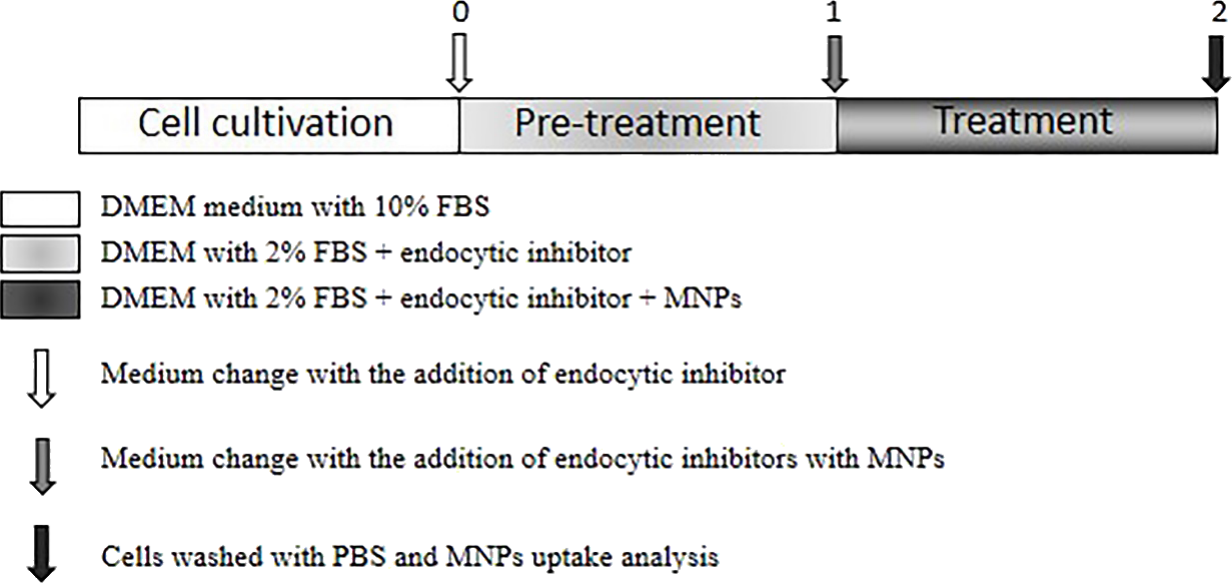
Scheme of cell exposure to endocytic inhibitors and surface-modified MNPs. At 75% to 80% confluence, cells were pre-treated (time 0) with endocytic inhibitor for 1 h in medium with 2% FBS, then (time 1) fresh medium with PEG-SO-MNPs or BSA-SO-MNPs in the presence of the same inhibitor was added for another period of 1 h. Cell exposure was finished by sucking off the culture medium (time 2) and washing the cells twice with phosphate-buffered saline (PBS).

After reaching exponential growth, cells were pre-treated with inhibitors of endocytosis or cytoskeleton dynamics for 1 h. Because endocytosis is a very fast process, the inhibitors are usually added to the culture cell media only for a short period of 1–2 h [68]. After pre-treatment, the medium was removed and a fresh medium containing the particular inhibitor and surface-modified MNPs was added to the cells for an additional time of 1 h. Cell exposure was finished by sucking off the culture medium and washing the cells twice with phosphate-buffered saline (PBS). An illustration of the treatment is shown in Figure 4.

The final concentrations of individual inhibitors of endocytosis or cytoskeleton dynamics were prepared freshly before use from the stock solutions. The final concentrations were prepared by dilution in a culture medium with 2% FBS. The stock solutions of chlorpromazine (CPZ, 5 mM) and methyl-β-cyclodextrin (MBCD, 20 mM) were prepared in sterile distilled water and kept at 4 °C. The stock solutions of monodansylcadaverine (MDC, 30 mM), filipin (F, 2 mM), nystatin (N, 5 mM), and dynasore (Dyn, 15.5 mM) were prepared in DMSO and kept at 4 °C. Nocodazole (Noc, 16.6 mM) was dissolved in DMSO, split to aliquots, and kept at −20 °C. The final concentration of DMSO never exceeded 0.52% (v/v). At this concentration, no cytotoxic and genotoxic effects were detected in A549 cells [69]. The final concentrations of the individual inhibitors are shown in Table 2. Control cells were treated with fresh culture medium and cells exposed only to MNPs for 1 h were considered as a positive control. These concentrations were selected based on the cell viability determined by MTT assay (Supporting Information File 1, Figure S5 and Figure S6). All inhibitors were purchased from Sigma-Aldrich (Slovakia). The cells exposed to culture medium were used as negative control and cells exposed only to MNPs for 1 h were considered as a positive control. The concentration of surface-modified MNPs was expressed as mM (mmol/L) of magnetite to apply equal numbers of nanoparticles to A549 cells. The stock solutions of PEG-SO-MNPs (194 mM), BSA-SO-MNPs (90.6 mM), and RITC-BSA-SO-MNPs (81.74 mM) were kept at 4 °C. The final concentration of surface-modified MNPs was 2 mM. RITC-BSA-SO-MNPS were diluted in a phenol-free medium to avoid interference.

**Table 2:** Inhibitors of endocytosis and cytoskeleton dynamics.

Endocytic pathway/target molecule	Inhibitor	Concentration [μM]

clathrin-mediated endocytosis (CME)	chlorpromazine (CPZ)	25
	monodansylcadaverine (MDC)	150
dynamin GTPase	dynasore (Dyn)	80
caveolin-mediated endocytosis (CavME)	filipin (F)	5
	nystatin (N)	20
lipid rafts	methyl-β-cyclodextrin (MBCD)	100
microtubules	nocodazole (Noc)	25

### MTT assay

The cytotoxicity of MNPs and endocytic inhibitors in A549 cells was assessed by the 3-(4,5-dimethylthiazol-2-yl)-2,5-diphenyltetrazolium bromide (MTT) assay following the protocol by Mosmann [70] with minor modifications. In brief, cells were plated in plastic 96-well plates at a density of 4 × 10^3^ cells per well. The photometric evaluation (at 540 nm excitation and 690 nm emission wavelengths) was carried out using a Multiskan Multisoft plate reader (Labsystems, Finland) and Genesis software provided by the manufacturer. As the color of MNPs is dark brown and may interfere with the spectrophotometry readings, the net readings were corrected according to Häfeli and co-workers [71].

### Time-lapse imaging of cells

Exponentially growing A549 cells were seeded on a 24-well tissue plate, at a density of 5 × 10^4^ cells per well. After reaching exponential growth, they were exposed to MNPs in the presence or absence of inhibitors as described above. After the treatment, cells were washed with PBS and post-cultivated in fresh DMEM supplemented with 10% FBS. Phase-contrast images were taken using the IncuCyte ZOOM™ Live Content Imaging System (Essen BioScience, Hertfordshire, UK) at 2 h intervals. Cell morphology and confluence after exposure to inhibitors were monitored using the IncuCyte ZOOM 2013A software as recommended by the manufacturer.

### Real-time RT-PCR (qRT-PCR)

Total RNA was isolated from cells using the phenol-chloroform method (TRIzol, Invitrogen, Carlsbad, CA) as recommended by the manufacturer and then purified and treated with DNase I. RNA concentration and purity were measured on a spectrophotometer (NanoDrop, Thermo Scientific). Degradation of RNA was excluded by electrophoresis. The cDNA was prepared using the RevertAid First Strand cDNA kit (Thermo Fisher Scientific) using 1 µg of total RNA according to the protocol recommended by the manufacturer. Gene expression was measured by semi-quantitative real-time PCR using SYBR Green dye (Maxima SYBR Green qPCR Master Mix kit) and appropriate primers, CLHC forward primer (5′-CCTAAACACCTCAACGATGAC-3′), CLHC reverse primer (5′-GTAAAACCAGTATTTCGTCAC-3′), Cav1 forward primer (5′-ACAATCGCTGGAAACAGAGT-3′), Cav1 reverse primer (5′-TGCAGGAGTTCTTCAGCCAAT-3′), GAPDH forward primer (5′-GCCAAAAGGGTCATCATCTC-3′), and GAPDH reverse primer (5′-CTAAGCAGTTGGTGGTGCAG-3′), on a CFX96TM Real-Time PCR Detection System cycler (Bio-Rad). Specifically, samples were denatured at 95 °C for 10 min, and the quantification program had 40 repeats (30 s annealing at 60 °C, 30s amplification at 72 °C). GAPDH was used as a “housekeeping” gene for the normalization of data.

### Western blotting

Cells were lysed in buffer containing 50 mM Tris/HCl, pH 7.4, 150 mM NaCl, 1% Triton X-100, and 1 mM EDTA, supplemented with protease inhibitor mix (Serva, BioTech, Ltd., Slovakia). After determining the protein concentration using the Bradford assay, equal amounts of proteins were boiled in Laemmli buffer and separated on a 10% polyacrylamide SDS-PAGE gel. After transfer of proteins onto nitrocellulose membranes the following primary antibodies were used: anti-Clathrin Heavy Chain (P1663) (#2410), anti-Caveolin-1 (#3238), anti-Phospho-Caveolin-1 (Tyr14) (#3251), anti-Dynamin I/II (#2342) (Cell Signaling Technology, BioTech Ltd., Slovakia), and anti-GAPDH (Sigma-Aldrich, Lambda Life, Ltd., Slovakia). Secondary peroxidase-labeled donkey anti-rabbit IgG (GE Healthcare, Germany) antibodies were visualized with Luminol and coumaric acid (Sigma-Aldrich, Slovakia).

### Atomic absorption spectrometry (AAS)

AAS was adapted to quantify the internalized amount of MNPs. All samples were analyzed twice by flame atomic absorption spectrometry for Fe. Cells were seeded on a Petri dish (100 mm diameter) at a density of 1 × 10^6^ cells per plate. At 75% to 80% confluence, they were exposed to MNPs in the presence or absence of inhibitors as described above. After the treatment, the number of cells was calculated, cells were harvested and digested using HNO_3_ (500 μL) in an ultrasonic bath at 85 °C for 2 h. The digests were then diluted with 2% HNO_3_ in deionized water. The instrumental parameters for Fe determination were set as follows: wavelength 248.3 nm, slit width 0.2 nm, flame type: acetylene–air, flow: 2.0 L/min for acetylene and 13.5 L/min for air, deuterium background correction, method of the calibration curve in the range 0.1–10 mg/L. The limit of detection (LOD) and limit of quantification (LOQ) for the AAS instrument were 0.0015 mg/L and 0.0049 mg/L, respectively. LOD and LOQ for the AAS method were 0.0074 mg/L and 0.0245 mg/L, respectively.

### Generation of stable clathrin-GFP expression cell line

Plasmid CLLCb-pEGFP (clathrin light chain B fused to enhanced green fluorescent protein) and vector pcDNA 3.1 (+) with neomycin resistance were transfected into A549 cells in a ratio of 7:1 using GeneCellin™ (Bio Cell Challenge) kit according to the manufacturer’s instructions. After 24 h, transfected A549 cells were selected in DMEM containing 10% FBS and 800 µg/mL geneticin G-418. Plasmid CLLCb-pEGFP was kindly provided by Prof. Ernst J. Ungewickell (Department of Cell Biology, Centre of Anatomy, Hannover Medical School, Germany) and vector pcDNA 3.1 (+) was kindly provided by Katarina Luciakova, DSc. (Cancer Research Institute, Biomedical Research Center SAS, Bratislava, Slovakia). Single-cell clones were selected and amplified by dilution cloning in 6-well plates.

### Immunofluorescence staining

Cells were grown on glass coverslips in 48-well plates overnight before incubation with Alexa Fluor 594–Transferrin conjugate (25 µg/mL) or cholera Toxin B subunit–FITC conjugate (5 mg/mL) at 37 °C for 1 h in medium supplemented with 2% FBS. In the case of the co-localization study, a phenol-free medium was used to avoid interference. FITC–clathrin A549 cells were pre-treated with inhibitor for 1 h and then treated for 1 h with RITC-BSA-SO-MNPs in the presence of the same inhibitor for another period of 1 h in phenol-free medium. Treatment was finished by three washes with PBS before fixation in 4% paraformaldehyde at room temperature (RT) for 20 min. Then, cells were permeabilized in 0.05% Triton X-100/PBS at RT for 15 min, washed with PBS, and blocked in 3% BSA/PBS at RT for 30 min. For F-actin staining, the coverslips were incubated with Alexa Fluor 488 Phalloidin or Alexa Fluor 546 Phalloidin (Life Technologies, Thermo Fisher Scientific) (dilution 1:500) at 37 °C for 1 h. After washing with PBS, 4,6-diamide-2-phenylindole (DAPI) (dilution 1: 500) was added for nuclear staining (at 37 °C, 15 min). Finally, after washing with PBS and sterile water, the coverslips were mounted on glass slides with mounting medium (Sigma-Aldrich). Images were obtained with a fluorescent microscope (Axio Imager, Zeiss) using ISIS software from the company MetaSystems GmbH (Altlussheim, Germany) at 630× magnification.

### Statistical analysis

Data are given as mean values ± SD. The differences between control cells and treated cells were evaluated by Student´s t-test and one-way analysis of variance (ANOVA). The threshold of statistical significance was set at *p* < 0.05.

## Supporting Information

File 1 Expression of clathrin and caveolin, cytotoxicity of MNPs and endocytic inhibitors, time-lap imaging and fluorescent microscopy of A549 cells.
