# Supplementary material for: Differences in surface chemistry of iron oxide nanoparticles result in different routes of internalization
Source: Beilstein J Nanotechnol. 2021 Mar 23;12:270–81. doi: 10.3762/bjnano.12.22 (PMC8008094; doi:10.3762/bjnano.12.22)
Supplement: File 1 — Expression of clathrin and caveolin, cytotoxicity of MNPs and endocytic inhibitors, time-lap imaging and fluorescent microscopy of A549 cells. [file Beilstein_J_Nanotechnol-12-270-s001.pdf]

BEILSTEIN JOURNAL OF NANOTECHNOLOGY

Supporting Information

for

Differences in surface chemistry of iron oxide nanoparticles result in different routes of internalization

Barbora Svitkova, Vlasta Zavisova, Veronika Nemethova, Martina Koneracka, Miroslava Kretova, Filip Razga, Monika Ursinyova and Alena Gabelova

Beilstein J. Nanotechnol. **2021,** *12,* 270–281. doi:10.3762/bjnano.12.22

Expression of clathrin and caveolin, cytotoxicity of MNPs and endocytic inhibitors, time-lap imaging and fluorescent microscopy of A549 cells

License and Terms: This is a supporting information file under the terms of the Creative Commons Attribution License (https://creativecommons.org/licenses/by/4.0). Please note that the reuse, redistribution and reproduction in particular requires that the author(s) and source are credited and that individual graphics may be subject to special legal provisions.
The license is subject to the *Beilstein Journal of Nanotechnology* terms and conditions: (https://www.beilstein-journals.org/bjnano/terms)

Expression of the key proteins involved in endocytosis in A549 cells

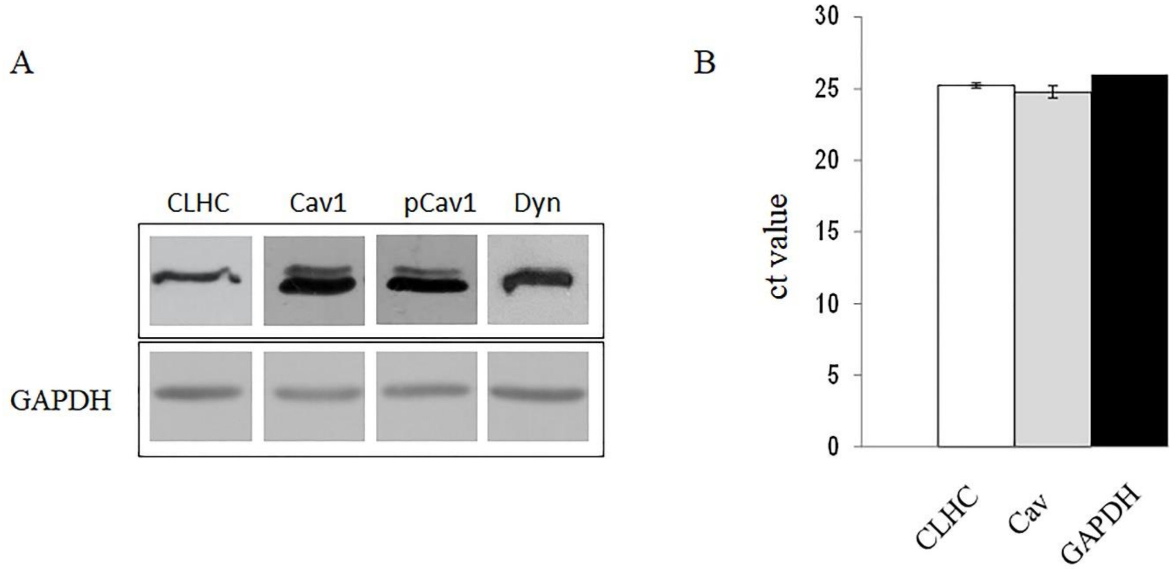

Figure S1: Expression of dynamin, clathrin, and caveolin in A549 cells. A – Western blotting, B – RT-PCR. Dyn – dynamin (M_w = 100 kDa), CLHC – clathrin heavy chain (M_w = 190 kDa), Cav1 – caveolin 1 (M_w = 21, 24 kDa), pCav1 – phospho-caveolin 1 (M_w = 23, 25 kDa), and GAPDH – glyceraldehyde 3-phosphate dehydrogenase (M_w = 36 kDa, housekeeping gene/protein).

Real-time phase-contrast images of A549 cells after exposure to surface-modified MNPs and endocytic inhibitors

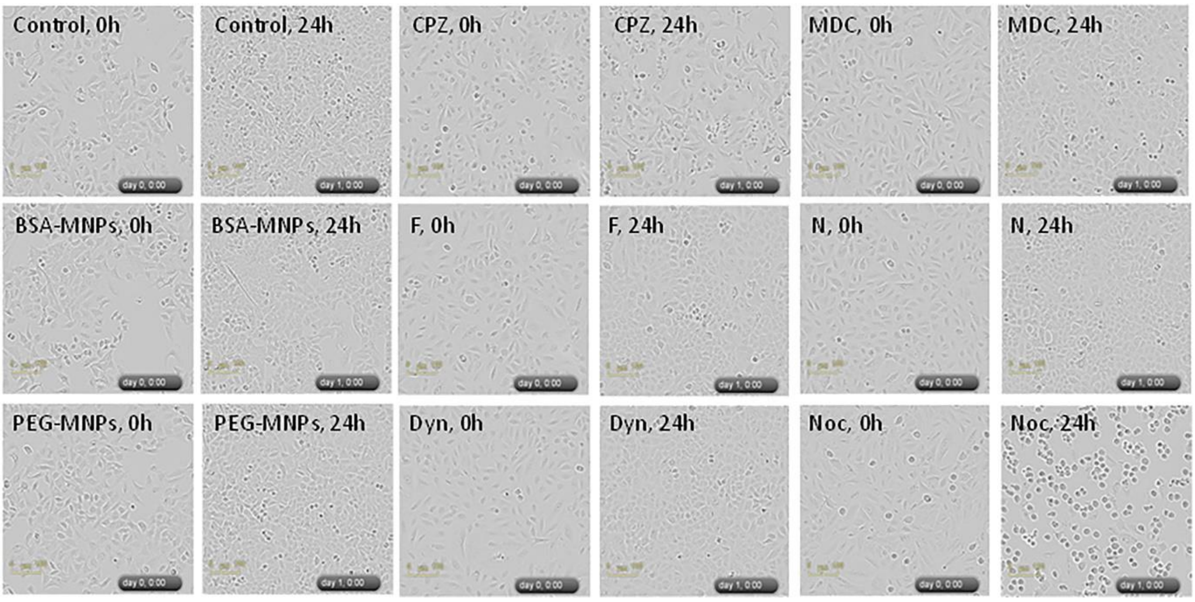

Figure S2: After treatment with MNPs (1 h) and endocytic inhibitors (2 h), cells were post-cultivated in fresh medium and screened for 24 h using the IncuCyte ZOOM™ Live Content Imaging System (Essen BioScience). CPZ – chlorpromazine, MDC – monodansylcadaverine, F – filipin, N – nystatin, Dyn – dynasore, and Noc – nocodazole. Scale bars represent 100 µm.

The effect of endocytic inhibitors on the internalization of positive controls: transferrin and cholera toxin

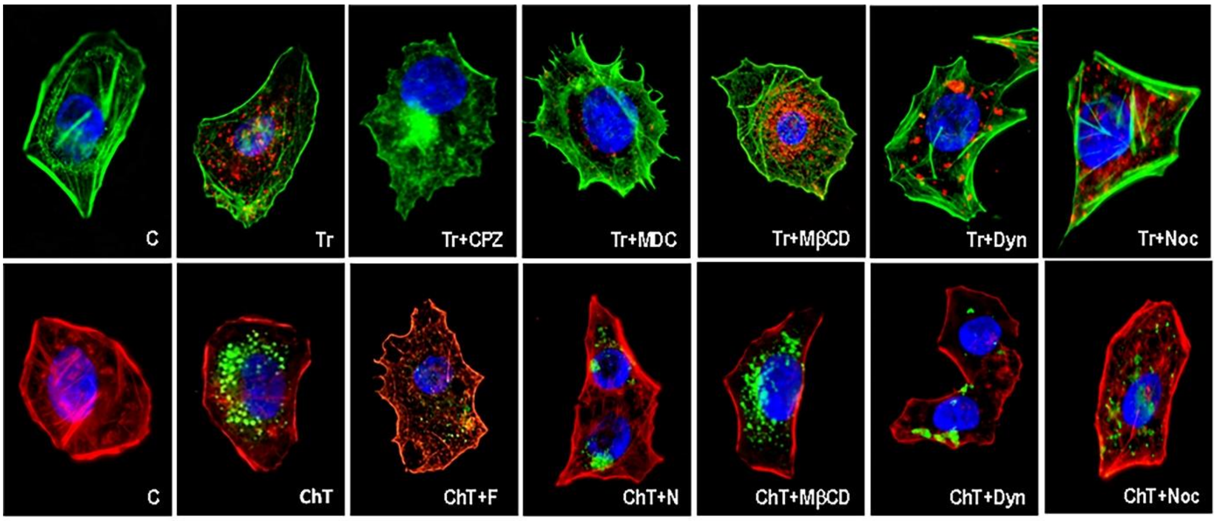

Figure S3: The effect of endocytic inhibitors on the internalization of positive controls: Tr (Alexa Fluor 594–Transferrin conjugate), positive control for clathrin-mediated endocytosis (CME), ChT (cholera Toxin B subunit–FITC conjugate), positive control for caveolin-mediated endocytosis (Cav1). Inhibitors: C – control, CPZ – chlorpromazine and MDC – monodansylcadaverine (CME), F – filipin, N – nystatin, MβCD – methyl-β-cyclodextrin (CavME/lipid raft), Dyn – dynasore, and Noc – nocodazole (inhibitor of microtubules). Tr – red, ChT – green, nucleus – blue (DAPI, 4,6-diamide-2-phenylindole), F-Actin – Alexa Fluor Phalloidin 488 (green) or Alexa Fluor Phalloidin 546 (red); magnification 630×.

Stable expression of green fluorescent protein tagged with clathrin light chain (CLLCb-GFP)

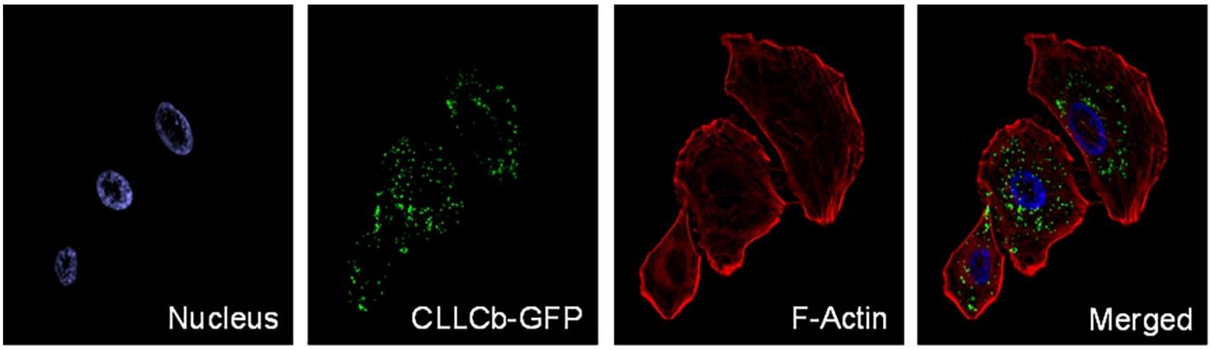

Figure S4: Stable expression of green fluorescent protein (GFP)-tagged clathrin light chain (CLLCb-GFP) in A549 cells. Blue – nucleus (DAPI, 4,6-diamide-2-phenylindole), green – CLLCb-GFP, and red – F-actin (Alexa Fluor Phalloidin 546); magnification 630×.

Cytotoxicity of surface-modified MNPs assessed by MTT assay

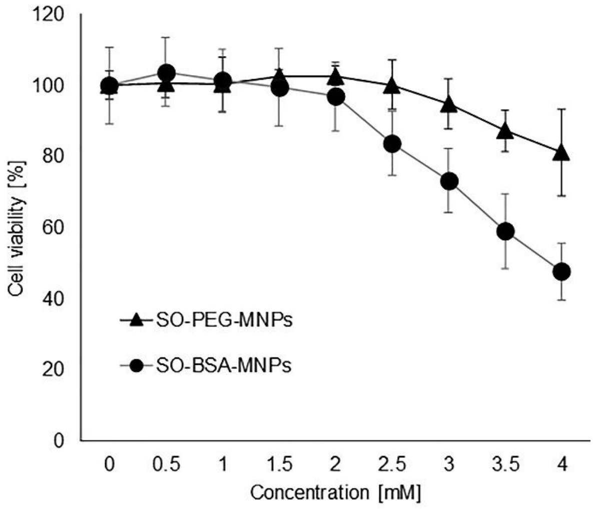

Figure S5: A549 cells were exposed to different concentrations of BSA-SO-MNPs and PEG-SO-MNPs for 1 h. Data are given as mean values ± SD from at least two independent experiments with eight parallel measurements.

Cytotoxicity of endocytic inhibitors assessed by MTT assay

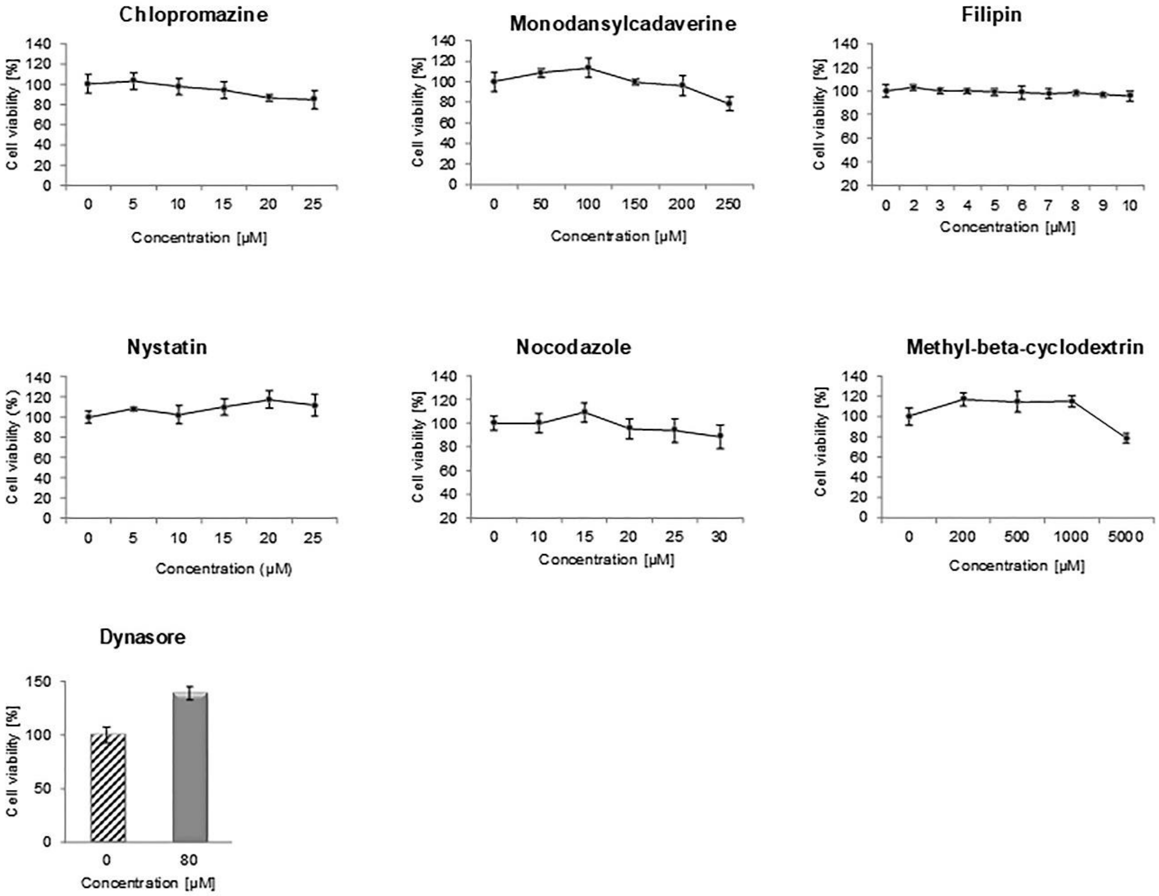

Figure S6: A549 cells were treated with various inhibitors of endocytosis for 2 h. Data are given as mean values ± SD from at least two independent experiments with eight parallel measurements.
